# Thoughts of Death or Self-injury, Non-same-day ART Initiation, and 2-year Incidence of Disengagement from Care Among People Entering HIV Care in Cameroon

**DOI:** 10.1007/s10461-026-05149-8

**Published:** 2026-06-05

**Authors:** Lindsey M. Filiatreau, Junu Rana Magar, Peter Vanes Ebasone, Morgan Shields, Anastase Dzudie, Marcel Yotebieng, Milton Wainberg, Angela M. Parcesepe

**Affiliations:** 1 School of Public Health, Washington University, Office 3123, 4300 Duncan Avenue, St. Louis, MO 63110, USA; 2 Clinical Research Education Networking and Consultancy, Yaoundé, Cameroon; 3 Department of Medicine, Albert Einstein College of Medicine, Bronx, NY, USA; 4 Department of Psychiatry, Columbia University and New York State Psychiatric Institute, New York, NY, USA; 5 Department of Maternal and Child Health, Gillings School of Global Public Health, University of North Carolina at Chapel Hill, Chapel Hill, NC, USA; 6 Carolina Population Center, University of North Carolina at Chapel Hill, Chapel Hill, NC, USA

**Keywords:** HIV, Self-harm, Self-injury, Depression, Suicidal ideation, Anti-retroviral therapy, Cameroon

## Abstract

Thoughts of death or self-injury and the clinical implications of such thoughts remain largely underassessed among people with HIV (PWH) in Africa. As strong predictors of suicidal ideation and death by suicide, it is paramount to understand these risk indicators, particularly in populations with heightened susceptibility to poor mental health. We aimed to characterize thoughts of death or self-injury (i.e., self-injurious thoughts) and their relationship with non-same-day (i.e., delayed) anti-retroviral treatment (ART) initiation and longitudinal disengagement from clinic in a cohort of PWH newly entering HIV care in Cameroon. We conducted structured interviews with PWH aged 21 + initiating clinical care between June 2019 and March 2020. Clinical records were used to ascertain ART initiation date and disengagement from the clinic across two years following care initiation. Log binomial regression was used to estimate the association between self-injurious thoughts at care initiation and delayed ART initiation. A Fine and Gray sub-distribution proportional hazards model was used to quantify the cumulative incidence of disengagement from the clinic (i.e., a gap in clinic visits > 183 days) and differences in these estimates across groups. Of 426 enrolled individuals, seventy-one (16.7%) endorsed self-injurious thoughts at entry into care, 24 (33.8%) of whom had *active* thoughts of self-injury. Self-injurious thoughts at entry into care were positively associated with delayed ART initiation (adjusted prevalence ratio = 1.8; 95% CI: 1.2, 2.9) and cumulative incidence of disengagement from clinic, though differences in disengagement were statistically non-significant. Interventions are urgently needed to support the mental health and well-being of PWH entering care and improve downstream HIV treatment outcomes.

## Introduction

During the early stages of implementation, same-day anti-retroviral therapy (ART) initiation policies (i.e., ‘Treat All’ policies) were widely heralded as the key to an AIDS-free generation [1–3]. While global implementation of such guidelines have yielded improvements in early-stage HIV care continuum outcomes, specifically reducing the time from diagnosis to treatment initiation [4, 5], evidence is mixed regarding the impact of such guidelines on downstream care outcomes (e.g., sustained engagement in care) [6, 7]. Other policies and programs, such as multi-month ART prescribing, peer support or navigation, community-based medication pickup programs, or population-specific clinics, may be needed to achieve optimal retention and viral suppression over time [8–12]. Indeed, one study from South Africa showed retention in care one year following linkage among those diagnosed with HIV before and after implementation of same-day treatment initiation policies were similar, highlighting that same-day treatment initiation policies alone are insufficient for improving long-term outcomes and ultimately ending the HIV epidemic [6].

In much of Africa, estimates of ART use, retention in care, and viral suppression continue to lag behind global targets even in the era of same-day treatment initiation policies; this is particularly true in populations with comorbid health conditions [13–15]. In Cameroon specifically, while the incidence of HIV has declined steadily over the past two decades, from 1.8 per 1,000 population in 2010 to 0.5 per 1,000 population in 2024, just 82% of people (74% of men) living with HIV were virally suppressed in 2024 despite free treatment across all levels of the health system since implementation of Universal Test and Treat in 2016 [16]. Moreover, in a study comparing viral load across twelve sub-Saharan African countries, Cameroon had the highest mean log_10_ viral load for both males and females, suggesting significant progress remains to achieve optimal treatment outcomes in this setting specifically [17].

Same-day ART initiation care models presume patients are psychologically ready to start lifelong treatment at the time of diagnosis or entry into care with HIV [4]. However, in practice, many people newly diagnosed with HIV experience acute psychological distress (e.g., heightened depressive symptoms and, in severe cases, thoughts of death, self-injury, or suicide) in response to their diagnosis or other difficult life circumstances (e.g., loss of a parent) that are common among people with HIV, particularly in Africa [18–21]. Such feelings may impact an individual’s readiness to start ART, especially if they believe life is no longer worth living [22]. Indeed, evidence from the continent demonstrates that psychological distress at or soon after HIV diagnosis is common and consequential [23]. For example, in one study from Ethiopia, the odds of psychological distress were significantly higher among people initiating ART who were newly diagnosed with HIV compared to others and among those who initiated ART late compared to others [24]. Importantly, psychological distress at the point of entry into HIV care may also hinder sustained engagement in care and other downstream care continuum outcomes (e.g., viral suppression), even among those who initiate treatment the same day they are engaged in care [24–26].

Suicidal ideation is a severe symptom of psychological distress associated with extreme negative health outcomes, including death by suicide [27–29]. Evidence shows people with HIV are particularly susceptible to these manifestations of distress, with a recent systematic review suggesting the risk of death by suicide among people with HIV is approximately 100 times the risk observed in the general population [30]. While these alarming statistics point to a clear need for more robust mental health screening, referral, and treatment systems, resource constraints, mental health-related stigma, limited infrastructure, and the criminalization of suicide have precluded the development of such systems in much of Africa [31–33]. In these settings, including Cameroon, integrating mental health services into more robust chronic care systems, such as those for HIV, may be particularly critical to closing the vast mental health treatment gap and preventing excess death by suicide among people with HIV [34].

Better understanding the magnitude and consequences of symptoms indicative of suicidal ideation among people entering HIV care can inform the design of robust, integrated mental health and HIV care delivery systems. Such systems can subsequently improve the health and well-being of people with HIV, and, most critically, prevent death by suicide in this population. To address existing gaps in the literature, this study aimed to characterize the prevalence and severity of thoughts of death or self-injury (i.e., “self-injurious thoughts”)—indicators of suicidal ideation and death by suicide [35, 36]—among people with HIV newly enrolling in HIV care in Cameroon. We also aimed to quantify the relationship between self-injurious thoughts and short- and longer-term HIV care continuum outcomes, specifically, delayed ART initiation and 2-year disengagement from clinic, to shed light on HIV-related implications of symptoms of psychological distress.

## Methods

### Setting

This study was conducted at three urban, publicly funded HIV treatment centers in Cameroon. Two clinics are in regions that have experienced unrest—the Northwest and Southwest Regions, while one is in the Centre Region, a comparatively more stable setting. Each of these facilities was purposively selected due to their participation in the International epidemiology Databases to Evaluate AIDS (IeDEA) Consortium [37] and, thus, demonstrated research capacity.

### Study Design and Population

This is a secondary analysis; the study design and methods have been previously described in detail in the main papers resulting from this work [38–42]. Briefly, any person with HIV aged 21 years or older who was initiating HIV care at any of the three included public HIV treatment centers in Cameroon from June 2019 to March 2020 was approached for enrollment into a prospective cohort. Individuals recruited from these health care facilities completed structured interviews with a trained research assistant at study enrollment (which occurred on or near an individual’s first HIV care visit) and at a single follow-up study visit that occurred approximately one to two years later. Survey data from the follow-up study visit were not used in the current analyses. Electronic health record data, including information on patient follow-up visits, official transfers, and deaths, were extracted by study staff for all enrolled participants at the end of study follow-up. Participants’ baseline survey data and longitudinal clinic data were linked using unique participant identification numbers.

### Measures

#### Outcomes: Timing of ART Initiation and Disengagement from HIV Care Clinic

Longitudinal clinic visit data, extracted at the end of study follow-up, were used to ascertain non-same-day (i.e., delayed) ART initiation and disengagement from clinic. Delayed ART initiation was defined as having a documented ART prescription after the first documented clinic visit for HIV care services. Disengagement from clinic was defined as having a gap in documented clinic visits of at least 183 days. That is, individuals were considered disengaged from the clinic on day 184 following their most recent visit, a definition consistent with a 6-month gap in clinical care, or being 3 months late for routine visits scheduled on a quarterly basis [43–45]. Documented clinic transfers and deaths were considered competing events, as these events precluded the occurrence of disengagement from care at the individual’s original clinic.

#### Exposure: Recent Thoughts of Death or Self-injury

The Patient Health Questionnaire-9 (PHQ-9), which has been validated and widely administered in similar settings in Africa, was used to assess thoughts of death or self-injury within the prior two weeks [46, 47] at study baseline. Individuals who responded “several days,” “more than half the days,” or “nearly every day” to PHQ-9 question nine, which asks individuals how often they have been bothered by “thoughts that you would be better off dead, or of hurting yourself” in the prior two weeks were considered to have “self-injurious thoughts” and were asked a follow-up question to clarify if they had “any thoughts of hurting yourself in some way” in the last two weeks. Individuals who responded “not at all” to this follow-up question were considered to have *passive* self-injurious thoughts. Those who responded, “several days,” “more than half the days,” or “nearly every day” were considered to have *active* self-injurious thoughts and were asked a series of five additional follow-up questions to determine the severity of these thoughts.

Questions used to assess severity included: “have you made any plans or considered a method to harm yourself” (yes/no), “have you ever attempted to harm yourself” (yes/no), “have you thought you might actually attempt to hurt yourself in the near future” (yes/no), “have you told anyone that you were going to commit suicide, or threatened that you might do it” (yes/no), and “do you think there is any risk that you might hurt yourself before you see your doctor the next time” (yes/no). Individuals who responded “No” to each of these five questions were considered to have *active-low* self-injurious thoughts. Individuals who responded “Yes” to the last question were considered to have *active-acute* self-injurious thoughts, and others (i.e., those who responded yes to any of the first four questions above but no to question five) were considered to have *active-moderate* self-injurious thoughts. These questions have been previously used to assess the severity of thoughts of self-harm among people with HIV in Malawi [48].

### Covariates

#### Sociodemographic Factors

Sociodemographic factors of interest were captured through self-report at study baseline and included age (continuous), gender (male/female), relationship status (single/partnered), mobility (< 1 month away from home in prior 12 months/1 + months away from home in prior 12 months), employment (not working for pay/working for pay), and household hunger measured on a continuous scale with scores ranging from 0 to 6 and higher scores being indicative of more severe household hunger.

### Psychosocial Factors

#### Social Support

As previously described, social support was measured with four items from the Multidimensional Scale of Perceived Social Support [49]. Two items assessed social support from friends, and two items measured social support from family. Respondents denoted their level of agreement with each statement about support using a 5-point Likert scale (1 = strongly disagree to 5 = strongly agree). A total social support score was created by summing responses to all items, with higher scores indicative of greater support.

#### Life Stressors

As previously described [40], to measure exposure to stressful life events in the three months prior to study enrollment, we use a modified, 12-item version of the Life Events Survey [50–52]. Specifically, participants were asked about negative relationship changes (e.g., getting divorced or separated, estrangement from a family member, an increase in arguments with a partner), death or severe illness of a family member or close friend, work-related difficulties (e.g., unable to find work, losing job or income), motor vehicle accidents, physical attacks, threats on one’s life, robberies, hospitalizations, major new illnesses, and safety concerns in one’s neighborhood. These events were selected for their relevance to the study setting and were operationalized as a continuous measure of total number of reported events experienced (0–12).

As previously described [53], to measure exposure to potentially traumatic events across the life course, we used a modified, 12-item version of the Life Events Checklist for DSM-5. Events of interest included: seeing people hitting or harming a family member in childhood, childhood physical assault or abuse, childhood sexual assault or rape, physical assault or abuse in adulthood by an intimate partner, physical assault or abuse in adulthood from a non-intimate partner, sexual assault or rape in adulthood, seeing someone physically assaulted or abused, seeing someone seriously injured or killed, experiencing a natural disaster, experiencing a serious accident or fire, exposure to war, and losing a child through death. One additional question asked individuals to identify any other traumatizing event experienced during childhood or adulthood. Exposure to potentially traumatic events was operationalized as a continuous measure of total number of reported events experienced (0–13).

#### Concurrent Symptoms of a Mental or Substance Use Disorder

Heightened symptoms of depression, anxiety, post-traumatic stress disorder, and harmful alcohol use were measured at study baseline. As previously described [38, 39], depressive symptoms were measured using the Patient Health Questionnaire-9 (PHQ-9), with scores of 10 or greater considered of indicative of heightened depressive symptoms [54]. Anxiety symptoms were measured using the Generalized Anxiety Disorder-7 (GAD-7) with scores of 10 or greater considered indicative of heightened anxiety symptoms [55]. Symptoms of post-traumatic stress disorder (PTSD) were measured using the PTSD Checklist for DSM-5 (PCL-5), with scores of 31 or greater considered indicative of heightened PTSD symptoms [56]. Hazardous alcohol use was measured using the Alcohol Use Disorders Identification Test (AUDIT) with scores of 8 or greater for men and 7 or greater for women categorized as hazardous alcohol use [57]. Each of these measures has been previously used in Cameroon and in other African countries, including among people with HIV, with high reliability. These measures were operationalized dichotomously as any versus no concurrent heightened symptoms of a mental or substance use disorder, as we have done in prior work [58].

### Statistical Analyses

Descriptive statistics were used to characterize the population overall, recent thoughts of death or self-injury, and variation in such thoughts across population sub-groups. Log binomial regression was used to quantify the association between self-injurious thoughts and delayed ART initiation. Disengagement from care across the first two years following linkage to care was quantified by fitting Fine and Gray sub-distribution proportional hazards models and using the Breslow estimator to calculate the cumulative incidence of the outcome. In time to event analyses, date of linkage to care served as the origin and administrative censoring occurred on day 730 following linkage to care or on January 1st, 2022, the date of database closure. In each of our adjusted analyses, we controlled for clinic, gender, employment, social support, food insecurity, and prior exposure to potentially traumatic or stressful life events, confounders identified a priori using a directed acyclic graph (Supplemental Fig. 1) [59, 60] constructed from a review of prior literature of factors associated with thoughts of self-harm among African youth and adults [61]. Notably, we did not review literature among people living with HIV specifically, as some of these individuals were likely newly diagnosed with HIV. In secondary models, we also controlled for concurrent symptoms of heightened depression, anxiety, post-traumatic stress disorder, and harmful alcohol use.

### Ethical Considerations

Ethical approval for this study was received by the University of North Carolina at Chapel Hill and the National Ethical Committee of Research for Human Health in Cameroon. Written informed consent to complete structured interviews was received from all study participants. Individuals endorsing moderate to acute self-injurious thoughts were immediately escorted to a clinician for additional assessment and intervention. Those endorsing passive or active-low self-injurious thoughts were referred for follow-up with a designated clinic staff member.

## Results

A total of 426 individuals were enrolled. All enrolled individuals were included in the analytic dataset, of whom a majority were female (*N* = 250; 58.7%), working for pay (*N* = 275; 64.6%), and in a relationship (*N* = 249; 58.4%; Table 1). The median age of study participants was 37 (interquartile range or IQR: 30–44). Nearly 30% (*N* = 119; 28.1%) reported moderate to severe household hunger and 39% (*N* = 164) reported being away from home for more than one month in the prior year.

**Fig. 1:**
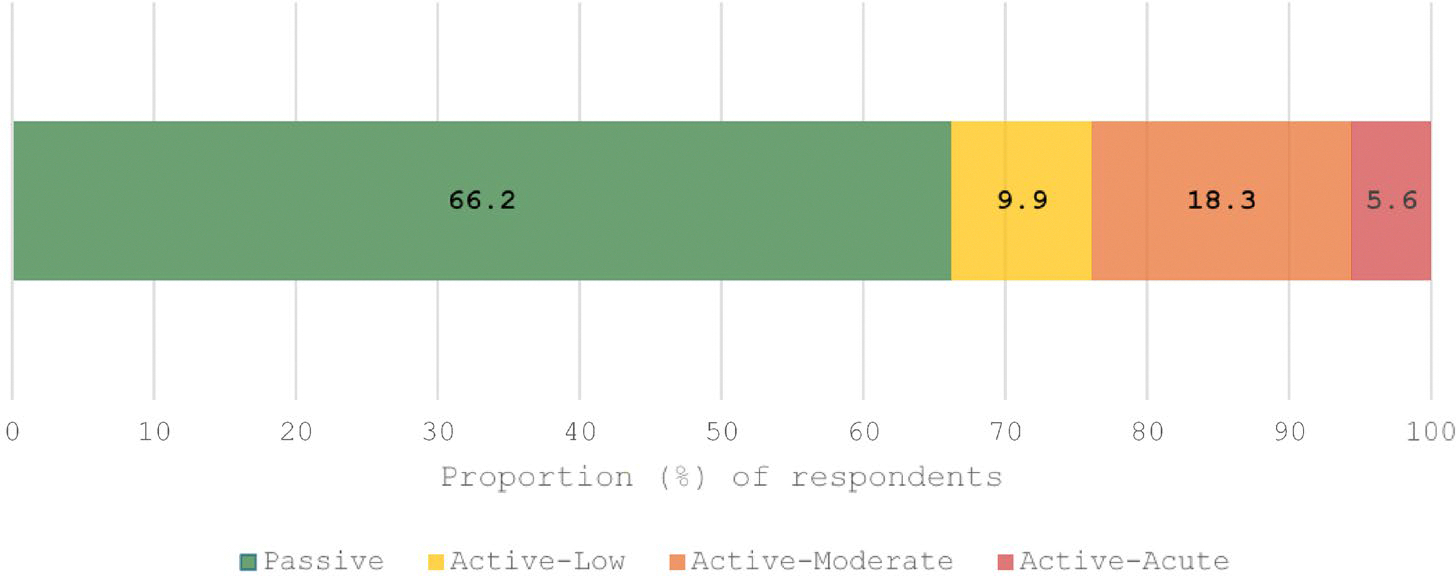
Distribution of the severity of self-injurious thoughts among individuals endorsing recent thoughts they’d be better off dead of or hurting themselves (*N* = 71)

Overall, 16.7% (*n* = 71) of study participants endorsed recent thoughts of death or self-injury. Of these 71 individuals, most (*n* = 47; 66.2%) endorsed *passive* self-injurious thoughts. Approximately one-quarter (*N* = 17; 23.9%) endorsed *moderate to acute active* self-injurious thoughts (Fig. 1), nine individuals had previously attempted to harm themselves, and four individuals reported there was a risk they might hurt themselves before they see their doctor again (Table 2). Individuals who were not in a relationship, those who spent more than one month away from home in the prior year, those endorsing moderate to severe household hunger, and those with heightened symptoms of a mental or substance use disorder were more likely to endorse self-injurious thoughts compared to their counterparts (Table 1). In addition, median number of stressful life events and potentially traumatic events were higher and median social support score was lower among those who endorsed self-injurious thoughts compared to others (Table 1).

Among 420 individuals with non-missing enrollment into care and ART start dates, 55 (13.1%) initiated ART after they enrolled into care as previously reported [38]. This comprised 27.5% (*N* = 19/69) of those endorsing recent self-injurious thoughts and 10.3% (*N* = 36/351) of those who did not endorse such thoughts (Table 3). The adjusted prevalence of delayed ART initiation among those endorsing recent self-injurious thoughts was 1.8 times (95% CI: 1.1, 2.9) that of others in this period (Table 3). Results from our secondary model, which additionally controlled for concurrent symptoms of heightened depression, anxiety, post-traumatic stress disorder, or harmful alcohol use, were similar [aPR 1.6 (95% CI: 1.0, 2.6)].

**Fig. 2:**
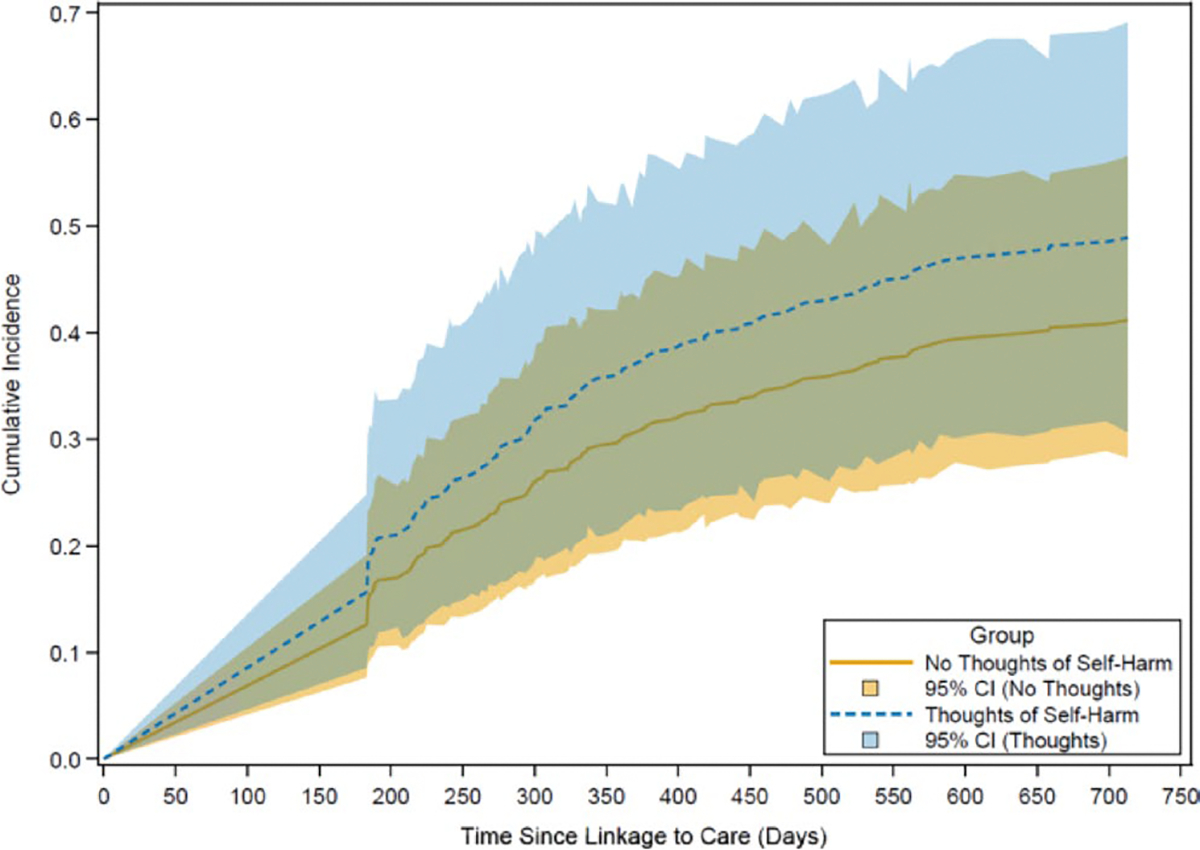
Cumulative incidence of disengagement from care adjusted for clinic, gender, employment, food insecurity, social support, and stressful and potentially traumatic events accounting for competing events (death and transfers) and stratified by endorsement of self-injurious thoughts at linkage to care in a cohort of people newly entering HIV care at three clinics in Cameroon between June 2019 to March 2020, (*N* = 411)

The cumulative incidence of disengagement from care was consistently higher among those who endorsed recent self-injurious thoughts at linkage to care compared to those who did not (Fig. 2), though these differences did not reach statistical significance. The adjusted cumulative hazard of disengagement across the first two years following linkage to care among those endorsing recent self-injurious thoughts at linkage to care was 1.3 (95% CI: 0.8, 2.0) times the hazard among others. Risk differences comparing estimates of disengagement among those with versus without recent self-injurious thoughts at engagement typically ranged from 3 to 8 percentage points while risk ratios were consistently 1.2 (Table 4).

## Discussion

This study aimed to characterize the occurrence and severity of recent thoughts of death or self-injury and quantify the association between self-injurious thoughts and HIV care outcomes in a cohort of people newly entering HIV care in Cameroon. We found that recent self-injurious thoughts were endorsed by 17% of the study population and were associated with delayed ART initiation. Though not statistically significant, disengagement from the clinic was consistently more common among those endorsing self-injurious thoughts at entry into care compared to their counterparts. These findings extend our other research that demonstrates the relationship between poor psychosocial well-being, namely heightened symptoms of depression, anxiety, and post-traumatic stress disorder, and sub-optimal HIV care continuum outcomes in this cohort of people entering HIV care in Cameroon [38, 39, 42, 62]. In the present analysis, we demonstrate how self-injurious thoughts, a strong indicator of suicidal ideation and death by suicide, are common and associated with failure to achieve early HIV care milestones among people entering HIV care in Cameroon.

While there is robust literature that highlights the disproportionate burden of mental health disorders among people with HIV compared to the general population [62, 63], suicidal ideation, death by suicide, and predictors of these extreme consequences of psychological distress, remain largely underassessed, particularly in Africa where mental health care infrastructure remains inadequate [64]. A 2021 meta-analysis exploring the prevalence of suicidal ideation and suicide attempts among people with HIV in Africa identified just 21 studies that reliably quantified the magnitude of suicidal ideation, none of which were conducted in Cameroon [65]. At 21.7%, the pooled prevalence of suicidal ideation in this meta-analysis was marginally higher than the prevalence of self-injurious thoughts observed in our study population, indicating a high degree of consistency in the occurrence of psychological distress symptoms among people with HIV in Cameroon and Africa more broadly. Jointly, the findings underscore the urgency of increased screening for early warning signs of such outcomes, particularly in chronic HIV care settings.

We also noted variation in the prevalence of self-injurious thoughts across population sub-groups in this cohort of people entering HIV care in Cameroon. Those who spent at least one month away from home in the prior year, younger individuals, those with moderate to severe household hunger, those with heightened symptoms of poor mental health and those with greater exposure to potentially stressful or traumatic events were more likely to endorse recent self-injurious thoughts compared to their counterparts. These findings are consistent with broader evidence that demonstrates mobility—including for work or marriage—can disrupt social support systems and introduce a host of life stressors [66, 67]; young people are often more susceptible to poor mental health as they navigate the emotional and social complexities of the transition to adulthood [68–72]; and unmet basic needs, particularly food insecurity, may amplify psychological distress among people with HIV [73, 74]. As one example, a 2023 systematic review which explored the relationship between food insecurity and suicidality found that the odds of both suicidal ideation and of suicidal planning were significantly greater among food insecure versus food secure individuals [75]. Mobility, younger age, and food insecurity have also been repeatedly linked to disrupted HIV care and suboptimal HIV care continuum outcomes, including ART non-adherence and viral non-suppression [76, 77]. Future research is needed to address the unique mental health challenges and the impacts of these challenges in highly mobile, younger, and food insecure populations specifically.

Despite the reliance of Treat-All policies on psychological readiness to begin lifelong therapy immediately after diagnosis [4], our results show respondents reporting self-injurious thoughts were less likely to start ART the same day they were linked to care when compared to their counterparts. These findings are consistent with prior work in Africa, which demonstrates that psychological distress is associated with delayed ART initiation [24, 78]. This includes our own research from this cohort, which found that those with heightened depressive and post-traumatic stress disorder symptoms were less likely to initiate ART on the same day they enrolled into care compared to their counterparts [38]. While we cannot disentangle if the decision to delay ART initiation was a provider, patient, or shared patient/provider decision, it is possible those experiencing self-injurious thoughts were also experiencing hopelessness (in living a healthy life in the future, or more broadly) [79], low-self efficacy, or lacked the motivation or confidence to commit to lifelong ART, particularly for those who were newly diagnosed [80]. It is also possible HIV care providers choose to postpone ART initiation in their patients who expressed extreme distress or difficulty during their first appointment to allow the patient time to process their diagnosis or need for lifelong treatment [81]. Other research, including findings from our prior work in this cohort, shows that other structural (privacy of services), community (e.g., stigma), relational (e.g., fear of disclosure), and individual-level factors (e.g., mobility, food insecurity) may also play a role in the timing of ART initiation [38, 81–83]. Additional research is warranted to disentangle if delays in ART initiation were patient or provider decisions, what motivates such decisions, and if such delays have significant impacts on downstream HIV or mental health care outcomes.

Though our findings were statistically non-significant, disengagement from the clinic was consistently more common among those endorsing self-injurious thoughts compared to others across the first two years following care engagement. Research has found that psychosocial factors, including limited social support, stigma, poor mental health, suicidal ideation, and hopelessness, are commonly reported reasons for disengagement from HIV care in low-resource settings [84, 85]. Mental health screening and intervention may be warranted across an individual’s care-seeking journey as poor mental health can impede both early and late-stage continuum outcomes.

Currently, Cameroon’s HIV treatment guidelines, like those in a vast range of settings, stop short of calling for integrated routine mental health screening in HIV prevention and care settings, despite the established link between HIV and poor mental health. This creates a missed opportunity to identify and link individuals experiencing poor mental health, including those experiencing more extreme distress such as thoughts of death or self-injury, or suicidal ideation to needed support networks, and, where available, evidence-informed interventions and mental health services. At best, these missed opportunities for mental health screening and intervention may result in lower quality of life among people living with HIV, and at worst, increased incidence of death by suicide. Importantly, consideration of the potential legal implications of identifying individuals experiencing self-injurious thoughts in African settings remains paramount. In Cameroon, where suicide and suicidal ideation are decriminalized, individuals should be screened for self-injurious thoughts at the time of diagnosis with HIV and throughout the early stages of the HIV-care cascade. Those endorsing such thoughts should be referred for mental health services where available and in instances where such services are unavailable, guided through efficacious safety planning and compassion therapy interventions that have been tested and implemented in other low-resource settings [86]. In African countries where suicide remains criminalized, decriminalization efforts are necessary before screening is widely implemented.

Importantly, a highly limited number of suicide interventions have been designed and tested in Africa specifically [87, 88]. Moreover, myriad barriers to implementation (e.g., shortage of trained personnel, competing priorities of clinic staff, and limited mental and physical health care infrastructure) and uptake (e.g., mental-health related stigma) of such interventions in settings like Cameroon have been well-documented [34, 87, 89–91]. However, potential solutions to such barriers, including co-creation of screening and treatment protocols with key stakeholders, training of paraprofessionals in mental health screening and safety planning interventions, and establishment of mental health consultation teams have also been detailed [89, 92]. Additional research and programmatic work are needed to adapt and scale efficacious suicide prevention interventions developed and implemented in similar settings and to design and test new approaches specifically for people living with HIV in Cameroon.

This study has limitations worth noting. First, as participants were recruited from three urban clinics in Cameroon, findings may not be generalizable to individuals in other settings. Also, all participants were initiating HIV care at one of the included study sites at the time of enrollment. Suicidal ideation may be different among people with HIV who have not yet been diagnosed or have not yet engaged in HIV care, limiting our understanding of the relationship between self-injurious thoughts and treatment initiation among people with HIV who have not yet initiated care. Further, self-injurious thoughts were assessed via self-reported responses to item 9 of the PHQ-9. Broadly, self-report measures are prone to social desirability bias; more specifically, item 9 of the PHQ-9 and thoughts of death and self-injury have been shown to be insufficient for consistently and reliably assessing suicidal ideation (sensitivity = 87.6%; specificity = 66.1%; negative predictive value = 97.2%; positive predictive value = 28.6%) [93, 94]. Still, the consistency of our results with other work supports the reliability of study findings. In addition to these limitations, time since first HIV diagnosis was unavailable, as were data regarding prior ART initiation and clinic visits at other care facilities. This limited our ability to ascertain if self injurious thoughts differed between those who were and were not newly diagnosed with HIV and those who may have previously initiated ART at another treatment facility. It also limited our ability to verify transfers and true disengagement from care. Finally, as mentioned above, we could not determine the reasons for delayed ART initiation and whether such decisions were motivated by the patient, provider, or both.

This work also has significant strengths. First, the longitudinal nature of the study facilitated an understanding of the relationship between self-injurious thoughts and short- and longer-term HIV care continuum outcomes. Second, we characterize not just the presence of self-injurious thoughts in the study population, but also the severity of these thoughts, a critical nuance that is frequently ignored or oversimplified in the literature. Finally, this research fills a substantial gap in our understanding of the relationship between self-injurious thoughts and HIV care continuum outcomes among people with HIV in Africa, given the dearth of evidence on this topic from this region.

## Conclusions

Thoughts of death or self-injury are common among people entering HIV care in Cameroon and are associated with delayed ART start and disengagement from HIV clinical care across the first two years following enrollment into care. Contextually appropriate screening and intervention are urgently needed to support the mental health and well-being of individuals entering HIV care in this setting. Such interventions may also yield improved short- and long-term outcomes across the HIV care continuum.

## Supplementary Material

Supplemental Material

**Supplementary Information** The online version contains supplementary material available at https://doi.org/10.1007/s10461-026-05149-8.

## Figures and Tables

**Table 1:** Sociodemographic characteristics of 426 people newly entering HIV care at three clinics in Cameroon between June 2019 to March 2020, stratified by self-reported thoughts of death or self-harm (i.e., self-injurious thoughts) in the prior two weeks

	Total (*n* = 426) *N* (col %)	Recent self-injurious thoughts (*n* = 71) *N* (row %)	No recent self-injurious thoughts (*n* = 355) *N* (row %)

Gender			
Male	176 (41.3)	24 (13.6)	152 (86.4)
Female	250 (58.7)	47 (18.8)	203 (81.2)
Age (median/IQR)	37 (30–44)	33 (27–43)	37 (31–45)
Relationship status			
Single	177 (41.6)	39 (22.0)	138 (78.0)
Partnered	249 (58.4)	32 (12.9)	217 (87.1)
Away from home > 1 month			
No	262 (61.5)	35 (13.4)	227 (86.6)
Yes	164 (38.5)	36 (22.0)	128 (78.0)
Employment status			
Not working for pay	151 (35.4)	30 (19.9)	121 (80.1)
Working for pay	275 (64.6)	41 (14.9)	234 (85.1)
Household hunger* (median/IQR)	0 (0–2)	1 (0–2)	0 (0–2)
No/low	304 (71.9)	40 (13.2)	264 (86.8)
Moderate/severe	119 (28.1)	30 (25.2)	89 (74.8)
Social support (median/IQR)	14 (12–16)	13 (11–14)	14 (12–16)
Exposure to potentially traumatic events (median/IQR)	4 (2–5)	5 (3–7)	4 (2–5)
Exposure to recent stressful life events (median/IQR)	3 (2–4)	4 (3–5)	3 (2–4)
Any heightened symptoms of a mental or substance use disorder			
No	253 (59.7)	16 (6.3)	237 (93.7)
Yes	171 (40.3)	55 (32.2)	116 (67.8)

* Missing: household hunger status n = 3; social support score n = 3; stressful life event score n=4; any heightened symptoms of a mental or substance use disorder n = 2

**Table 2:** Endorsement of recent self-injurious thoughts among 426 people newly entering HIV care at three clinics in Cameroon between June 2019 to March 2020

Severity assessment questions	*N* (% of study population; % of 71 endorsing recent self-injurious thoughts)

Thoughts that you would be better off dead, or of hurting yourself in prior two weeks	71 (16.7; 100.0)
Thoughts of hurting yourself in some way in prior two weeks	24 (5.6; 33.8)
Made any plans or considered a method to harm yourself in past month	11 (2.6; 15.5)
Ever attempted to harm yourself	9 (2.1; 12.7)
Might actually attempt to hurt yourself in the near future	6 (1.4; 8.5)
Told anyone you are going to commit suicide or threatened that you might do it in past month	6 (1.4; 8.5)
Think there is any risk that you might hurt yourself before you see your doctor the next time	4 (0.9; 5.6)

**Table 3:** Log-binomial regression results estimating the association between recent self-injurious thoughts and delayed ART initiation among a cohort of people newly entering HIV care in Cameroon between June 2019 to March 2020 (*N* = 426)

	Total population (*n* = 426) *N* (col %)	Same day ART initiation^a^ (*n* = 365) *N* (row %)	Delayed ART initiation^a^ (*n* = 55) *N* (row %)	(*n* = 420) uPR (95% CI)	(*n* = 411) aPR^b^ (95% CI)

No thoughts of self-harm in prior 2 weeks	355 (83.3)	315 (89.7)	36 (10.3)	–	–
Thoughts of self-harm in prior 2 weeks	71 (16.7)	50 (72.4)	19 (27.5)	2.7 (1.6, 4.4)	1.8 (1.1, 2.9)

a six individuals were missing ART start date and were therefore excluded from regression analyses

b adjusted for clinic, gender, employment, household hunger, social support, and exposure to potentially (lifetime) traumatic and stressful life events (past three months)

**Table 4:** Adjusted cumulative incidence of disengagement among individuals newly entering HIV care with and without (referent) self-injurious thoughts at three clinics in Cameroon and corresponding risk ratios and risk differences (*N* = 420)

	No past 2-week self-injurious thoughts (*N* = 355)	Past 2-week self-injurious thoughts (*N* = 71)		

Time (days)	Cumulative incidence (95% CI)	Cumulative incidence (95% CI)	Risk difference (95% CI)	Risk ratio (95% CI)

183	12.6 (7.7, 19.1)	15.7 (8.6, 24.8)	3.1 (−3.4, 11.1)	1.2 (0.8, 2.0)
365	30.2 (20.6, 43.3)	36.6 (22.0, 53.9)	6.4 (−7.8, 20.4)	1.2 (0.8, 1.7)
548	37.5 (25.6, 52.9)	44.8 (27.8, 64.8)	7.3 (−8.7, 22.5)	1.2 (0.8, 1.7)
730	41.1 (28.3, 56.6)	48.9 (30.6, 69.1)	7.7 (−8.6, 24.1)	1.2 (0.8, 1.7)
