# Supplementary material for: Thoughts of Death or Self-injury, Non-same-day ART Initiation, and 2-year Incidence of Disengagement from Care Among People Entering HIV Care in Cameroon
Source: AIDS Behav. Author manuscript; Available in PMC 2026 Aug 11. (PMC13459024; doi:10.1007/s10461-026-05149-8)
Supplement: Supplementary file 1 [file NIHMS2195465-supplement-Supplemental_Material.docx]

**SUPPLEMENTAL MATERIAL**

**Supplemental Figure 1. Directed acyclic graph for ascertaining minimally sufficient adjustment set (adjustment variables in red)**

Age

Employment

Prior traumatic events; stressful life events

Food insecurity

Gender

Mobility

Suicidal ideation

Delayed treatment initiation

Social support
